# 33149 Interventions and Education: What We Learned from the 'All Eyes on Us' Study

**DOI:** 10.1017/cts.2021.596

**Published:** 2021-03-30

**Authors:** Sara Kennedy, Sarah Koopman Gonzalez, Leslie Richards, Bridget Croniger, Gabrielle Blackshire, Erika Trapl, Jessica Cooke Bailey

**Affiliations:** Case Western Reserve University

## Abstract

ABSTRACT IMPACT: This study identifies potential areas for community and clinical interventions to improve eye and vision health. OBJECTIVES/GOALS: The ‘All Eyes on Us’ study sought to understand perceptions of and barriers to eye and vision care, of residents over the age of 40 in the Broadway/Slavic Village neighborhood in Cleveland, Ohio. The goal of this study was to identify potential areas for community and clinical interventions to improve eye health. METHODS/STUDY POPULATION: Residents of the Broadway/Slavic Village neighborhood, an ethnically diverse, low socioeconomic status, neighborhood in Cleveland, Ohio were recruited from, and with the assistance of, University Settlement, a nonprofit that has been providing services to the neighborhood since 1926. The project’s Community Advisory Board assisted with the development of a semi-structured interview guide over the course of three meetings. Sixty interviews were completed, 30 with self-identified European Americans and 30 with self-identified African-Americans, all over the age of 40. Two research team members coded the interview transcripts and a thematic analysis was conducted. RESULTS/ANTICIPATED RESULTS: Participants identified barriers to obtaining eye and vision care for themselves as well as perceived barriers for others, including transportation, cost, insurance status, clinic locations, lack of education around eye and vision care, fear, forgetfulness, and priority management. To encourage people to go to the eye doctor more often, participants mentioned strategies related to access issues including lowering the cost of exams, operating on a sliding scale, improving insurance coverage, transportation services, and having mobile units that deployed to specific neighborhoods or senior centers. Additionally, participants suggested education and increasing awareness about the importance of eye and vision care. DISCUSSION/SIGNIFICANCE OF FINDINGS: Participants in this study identified that accessibility to and awareness about eye health and eye care is an issue. Interventions to address both access issues such as location, cost, and insurance as well as those that increase education could increase engagement with eye and vision care.

